# European Correspondence

**Published:** 1868

**Authors:** L. P. Yandell

**Affiliations:** Paris, France


### ARTICLE IV.

PARIS, FRANCE, December 26th, 1867.

*Dr. John M. Johnson:*

DEAR SIR—For several days past I have been making a general tour of the Hospitals and private cliniques of Paris, in company with Professor Armstrong, your very clever countryman of whom I spoke in my last; and I hope the account which I am about to give you of some of the things I saw and heard may interest you, though I have little expectation of affording you much valuable information. Let me say, *in limine*, that there is one most remarkable and culpable picture in hospital management here, which I have observed in all the institutions that I have visited—namely, that small pox patients are not separated from the other sick, but lie side by side with the subjects of ordinary diseases, although its contagiousness is doubted by no one. Cholera patients, on the other hand, are isolated; while its communicability by contact is by no means a universally received opinion. Seeing pregnant women and little children thus constantly and cruelly exposed to this loathsome contagion, I asked, Why is it that small pox is brought in this way so near to those who may be subject to it? "Routine," was the reply of the Interne- It has been the custom from time immemorial, and it is tedious and troublesome to effect any change in the hospital regulations; for hospital affairs, like almost all other affairs in the Old World, are in the hands of a circumlocution office, through which business moves slowly. The old, according to the proverb, learn new tricks with difficulty.

On my last visit to the St. Louis I saw Hardy injecting corrosive sublimate into the arm of a patient with secondary syphilis by means of the hypodermic syringe. I am sorry I am not able to give the result of the treatment. At the Hotel Dieu a favorite treatment for ophthalmia neonatorum is the hourly injection of simple warm water into the eyes.

For acute rheumatism, laudanum on cotton batting, in which the part is well wrapped, is a practice much used here. Very little of the fluid is absorbed by the batting. The part is kept very warm, and local transpiration thus augmented. Doubtless, in chronic rheumatism, the remedies would act equally well.

A few days since I followed Maisonneuve through his wards, and afterwards attended his clinique in the hospital Amphitheatre. He treats erysipelas by blisters applied directly to the diseased surface. His doctrine is, that erysipelas depends upon a morbid agent in the blood, to which nature is endeavoring to expel, and by a blister we afford the ferment an opportunity of egress. He treats amputations by the exclusion-of-air plan. A gumelastic bag is fitted on to the stump, and from the closed end of the bag a tube is connected with an air-pump, and the air is thus drawn off. He has no case at present under treatment, and I have not seen the process applied. It is claimed for it that it prevents supparation; but this, I believe, is doubted. The method by acupressure is in every respect so far superior to it that I won't describe the plan of M. Maisonneuve more fully. He treats fractures by dressings with plaster of Paris. You have, for example, a fractured tibia or fibula; four or five pieces, or more, of cotton cloth a foot wide, and capable of reaching from above the knee to the foot, are saturated with a mixture of the plaster and water and then applied to the limb, having first folded each piece of cloth four times. One runs from above the knee in front down to the instep or toes. Then side pieces are adjusted—two or more, as you deem necessary. A roller bandage is then quickly applied from the toes up above the knee. In eight minutes the dressing is firm. You may then remove the bandage and apply a few adhesive strips where they may be needed to keep the splints in place. No cotton batting is used by Maisonneuve. In other hospitals the plaster of Paris dressing is applied with the many-tailed bandage, or ordinary roller, and padding is employed. The method of M. Maisonneuve strikes me as the preferable one; but I leave the question to be decided by the surgeons.

M. Maisonneuve is a short, big-bellied, pug-nosed man, and looks like an ugly, fat old woman. He speaks clearly and majestically, but not rapidly. At his clinique he operated for fistula in ano, hemorrhoids, and stricture, and removed a female mamma. In only the last of these operations did he administer chloroform. As a surgeon, he is slovenly and rough, and his patients wince as he approaches them.

While waiting for Dr. Jarjavy at the Hospital of the Cliniques, to go through the wards with him, a few days since, I took a hasty survey of the lying-in room, which is always open to students, and saw there a woman in the pangs of childbirth. On either side, at the patient's knees, stood a young female, apparently friends of the woman. Near by was the *Sage Femme.* The legs of the patient were flexed, and a blanket was thrown over them, but so as to leave exposed to view the whole process of parturition. As I entered the room, with several others, the young girls blushed slightly, and looked as if their modesty was a little shocked, but the soon-to-be mother showed no signs of embarrassment.

At La Charité, Gosselin is a favorite with students, and from fifty to sixty follow him daily in his rounds. Amongst the students, I was astonished to see a female, and on inquiry learnt that she is a country-woman of ours, from New York, or Boston. The young woman is not more than eighteen or twenty years of age, and is by no means ill-favored. French students are no respectors of even female persons, and their fellow-student in petticoats is pushed and crowded about by them just as though she had worn pantaloons. She seems to be an earnest student, but is lady-like and modest in her demeanor. I was glad to see that she stood back during the introduction of catheters, and the examination of gangrenous penises, cancerous testicles, etc. I wondor that she does not devote herself exclusively to Obstetrics. Both in London and in Paris there are women who stand high in the profession as Obstetricians, and the time will doubtless come when we in America shall confide that business entirely to their hands. We may never consent to female suffrage, but I expect to see the day when we shall allow the women to bring out all the voters, and their sisters.

M. Gosselin employs the silicate of potash instead of plaster of Paris, in dressing fractures. It is used of the consistency of syrup, and is put on like starch or the plaster. The objection to this material is that it requires two hours to acquire any firmness, and two days to become perfectly hard. On this account it seems to me inferior both to starch and the plaster of Paris.

I am charmed with Roger and Bouchest, at the Children's Hospital. Bouchest is fully six feet high, erect and graceful, and in his elasticity of manner reminded me of Dr. Gross. Roger is a diminutive, round specimen of a man, not more than five feet two, and the gentlest mannered man at the bed-side that I have ever seen. He patted, and fondled, and soothed his little patients as you see tender mothers do their sick children, and whenever he had occasion to disturb or fatigue one of the little sufferers by a more than usually tedious examination, he rewarded it with pennies. As a lecturer he is remarkably humorous, and constantly excites the laughter of his students. In the sick room he is soothing; in the lecture room he is sparkling.

I have heard lectures from Depaul and Jarjavy at the Hospital of the Cliniques, Maissonneuve Roger, and Gosslin at the Hotel Dieu, La Charité, and the Children's Hospital, and heard Bazin, Hardy, Tardieu, Bouchest, Vermueil, and others, at the bed-side. All are fluent speakers. Their ease and grace of manner make it a pleasant task for students to listen to their lectures. In point of utterance they are incomparably superior to their English brethren, who, certainly, of all the intelligent people I have yet encountered, have the poorest use of their mouths. The power of the English physicians is in their pens.

Of the Eye Cliniques, Desmarres' is the finest that I have seen. Desmarres is an exceedingly handsome young man, about thirty years old, a showy operator, and, like all his countrymen, a fluent talker. I saw him examine and prescribe for a hundred cases, and operate for three cataracts, in two hours. He everts the lids with his left hand alone, and does it almost as quickly as he could snap his fingers. Galayowski, a delicate little pole, earnest and clever as a teacher, and Liebreich, a sickly looking, but pushing, live young Frenchman, both have excellent Eye Cliniques..—Fanvell's Throat Clinique is an exceedingly fine one. He uses the Drummond light in his laryngoscopical examinations. I saw him remove two small polypi from the vocal cords. He is a good talker, and wonderfully polite and attentive. Besides these, there are many other Cliniques and Hospitals in Paris, of which I have not now time to speak, if my letter had not already grown too long.

Very truly yours,

L. P. Yandell, Jr.

